# Optimization of a Therapeutic Vaccine Candidate by Studying Routes, Immunization Schedules and Antigen Doses in HBsAg-positive Transgenic Mice

**DOI:** 10.5005/jp-journals-10018-1105

**Published:** 2014-07-28

**Authors:** H Trujillo, A Blanco, D García, F Freyre, J Aguiar, Y Lobaina, JC Aguilar

**Affiliations:** 1 Hepatitis B Department, Biomedical Research Unit, Center for Genetic Engineering and Biotechnology, Havana, Cuba; 2 Animal Facilities, Center for Genetic Engineering and Biotechnology, Havana, Cuba

**Keywords:** HBV, Parenteral, Nasal, Therapeutic vaccine.

## Abstract

Hepatitis B core antigens (HBcAg) and hepatitis B surface antigens (HBsAg) are the main structural antigens of hepatitis B virus (HBV). Both antigens are potent immunogens for experimental animals as well as in acutely infected patients. A novel formulation based on the combination of HBsAg and HBcAg has been developed as a therapeutic vaccine candidate, aimed at inducing an immune response capable of controlling the infection. An immunization schedule was conducted to evaluate the immunogenicity of this formulation after simultaneous immunization by the intranasal and parenteral routes using different schedules and doses. Humoral and cellular immune responses generated in blood and spleen were evaluated by engyme-linked immunosorbent assay (ELISA) and enzyme-liked immunospot (ELISPOT) assays respectively. A first experiment evaluated two groups of mice simultaneously immunized by intranasal (IN) and subcutaneous (SC) routes, one including alum by SC route and, in the other, the formulation was injected without adjuvant.

As a result, alum adjuvant did not increase the immunogenicity under the studied conditions. In fact, the group without alum induced the most potent immune response. The immune response was enhanced by combining IN and SC immunization compared to the SC route alone. In a second experiment, mice were immunized by different mucosal routes at the same time, and compared to the simultaneously (IN/SC) immunized groups. It was demonstrated that there is no improvement on the resulting immune response by using multiple routes of immunizations simultaneously; however, the increase of the antigen dose induced a superior immune response. Interestingly, the increase of antigen dose only by SC route did not favor the resulting immunogenicity. In conclusion, the use of HBsAg transgenic mice has proven useful to optimize the formulation, avoiding the unnecessary use of alum as adjuvant as well as provided information of the role of different mucosal immunization routes and antigen dose on the resulting immune response.

**How to cite this article:** Trujillo H, Blanco A, García D, Freyre F, Aguiar J, Lobaina Y, Aguilar JC. Optimization of a Therapeutic Vaccine Candidate by Studying Routes, Immunization Schedules and Antigen Doses in HBsAg-positive Transgenic Mice. Euroasian J Hepato-Gastroenterol 2014;4(2):70-78.

## INTRODUCTION

The infection by hepatitis B virus (HBV) is an important world health problem in spite of the very effective vaccines existing since more than 20 years ago. About two billion people evidence markers of infection in the past and more than 350 million are persistently infected. The chronic disease correlates with an increased risk of developing liver cirrhosis, hepatocellular carcinoma and other complications, such as portal hypertension and liver failure. As a consequence, one million people die each year worldwide.^1,2^

Hepatitis B core (HBcAg) and surface (HBsAg) antigens are the main structural antigens of HBV. Both antigens constitute potent immunogens for experimental animals as well as in acutely infected persons.^3^

The HBcAg is one of the immunodominant components in HBV infection.^4,5^ The strong immunogenicity of this antigen has been explained for its dual behavior as a T-cell dependent and independent antigen.^6^ The direct and potent activation of B cells, which functions as primary antigen-presenting cell (APC) for HBcAg, also explains the remarkable immunogenicity of HBcAg.^7,8^

The antibodies against the HBcAg appear early during the course of the HBV infection and they often persist for years after the recovery of the disease.^9^ In vaccine studies, the HBcAg has shown to be highly immunogenic even without adjuvants and has been studied as a carrier molecule for homologous and heterologous epitopes.^10,11^

The HBsAg is the antigen used in the commercial recom-binant HBV vaccine, which has been proven over the last 20 years to be a very safe and effective prophylactic vaccine against HBV infection.^12^

The use of the current preventive vaccines has been previously reported in the field of therapeutic vaccination against chronic hepatitis B (CHB).^13,14^ The therapeutic evaluations of vaccines based exclusively on HBsAg have resulted safe, but poorly effective. The use of new formulations, novel adjuvant strategies and routes of immunizations has been suggested to overcome the state of unresponsiveness developed by the chronic disease.^15,16^

Several studies support the use of HBcAg as the antigen of choice to increase the number of epitopes and the variety of the immune response, alone or in combination with other antigens, such as HBsAg.^16-18^

Recently, a novel formulation was developed, a therapeutic vaccine candidate for both, nasal and parenteral administration, comprising HBsAg and HBcAg. Preclini-cal and clinical studies have demonstrated a good safety and immunogenicity profile.^19,20^ However, the immunization route, schedule of immunization and antigen dose need to be optimized in order to improve the capacity of the formulation to overcome the immunological tolerance. One of the most suitable models to optimize such variables is the model of the HBsAg-transgenic (Tg) mice that simulates the state of immunotolerance found in CHB patients.

The objective of the present study is to evaluate the immunogenicity of the therapeutic vaccine formulation in HBsAg-Tg mice using different combinations of routes and dose levels as well as the assessment of the parenteral route of immunization with or without alum as adjuvant. In addition, the histopathological studies were conducted to evaluate the potential risk of subverting the immuno-genicity to a ‘self’ antigen.

## MATERIALS AND METHODS

### Antigens

The recombinant HBcAg particle of 183 amino acids was expressed in *Escherichia coli (E. coli)* and purified for immunization experiments. Recombinant HBsAg subtype adw2 was obtained from the production process of the Cuban hepatitis B vaccine Heberbiovac-HB (CGEB, Havana, Cuba). This HBsAg was expressed in the yeast *Pichia pastoris (P. pastoris).* Both particles were obtained and purified to a minimum of 95% purity. The formulations containing these antigens was obtained by simple mixture in phosphate buffer saline (PBS) (0.1 mol/l NaCl, 2 mmol/l KCl, 10 mmol/l, Na_2_HPO_4_, 1 mmol/l KH_2_PO_4_, pH 7.2) and named NASVAC. For groups immunized with aluminum hydroxide (alum), we employed the nasal vaccine candidate (NASVAC) formulation adsorbed to 1 mg/ml alum.

### HBsAg-Tg Mice and Immunization Schedules

The HBsAg-Tg transgenic mice (with Balb/c genetic background) were used for two immunization schedules. HBsAg-Tg mice were obtained at the Center for Genetic Engineering and Biotechnology (CIGB, Havana, Cuba) by genetic transfer to mice embryos as previously described.^21,22^

A total of 22 males and females, 9 weeks old HBsAg-Tg mice were immunized 25 times in the first experiment. Mice were divided into three groups of six animals (3♂ y 3♂) and 1 control group of four non-treated animals (2♂ and 2♀). Groups 1 and 2 were immunized with 5 μg HBsAg + 5 μg HBcAg IN and SC and group 3 received PBS (IN) and 10 gm HBsAg + 10 μg HBcAg (SC). The groups 1 and 3 received in the SC immunization after the adsorption of the antigens in 1 mg/ml aluminum hydroxide (Brenntag Biosector, Vedbaek, Denmark), referred in this work as alum. The SC administration was administered in a final volume of 100 μl (groups 1 and 2) and 200 μl (group 3). For the nasal route, mice were anesthetized by intraperitoneal (IP) administration of 30 μl ketamine (10 mg/ml), placed in a supine position and the immunogens dispensed slowly in 50 μl (25 μl/ nostril) using a pipette tip. In all cases, the formulation with the adjuvant was prepared the day before and stored at 4°C until inoculation. Immunizations were carried out with a 14 days interval and the sera were collected 10 days after 3rd, 5th, 7th, 10th, 13th, 15th, 17th, 19th, 21st, 23rd and 25th dose.

For the second experiment, 35 HBsAg-Tg mice were divided in five groups of six animals (3♂ and 3♀) and one control group of five animals (3♂ and 2♀) to receive 10 immunizations. The groups were inoculated as follow: (G1) 5 g HBsAg + 5 μg HBcAg (IN and SC), (G2) 20 μg HBsAg + 20 μg HBcAg (IN) and 5ug of each antigens (SC), (G3) 5 μg of each antigens (IN) and 20 μg HBsAg + 20 μg HBcAg (SC), (G4) 20 μg HBsAg + 20 μg HBcAg (IN and SC), (G5) 5 μg HBsAg + 5 μg HBcAg, simultaneously administered by the IN, oral, intrarectal (IR), sublingual (SL) and SC routes. Mice from group 6 were not treated and regarded as a negative control group. The formulations were administered in a final volume of 100 μl. The doses were given on days 0, 14, 28, 42, 56, 70, 84, 98, 112, 126, and blood extractions were carried out on days 38, 66, 94, 136.

### Biological Fluids

Blood samples were collected through the retro-orbital plexus and centrifuged at 12,000 × g for 10 mins (5415 C Eppendorf Centrifuge, Hamburg, Germany), and the sera were collected and stored at - 20°C until evaluation.

### Total IgG and Subclasses Response Evaluation by ELISA

Specific IgG against HBsAg and HBcAg were evaluated by indirect ELISA, as previously described.^23^ Briefly, high binding plates (Costar, USA) were coated with 100 μl of the specific antigen at 5 μg/ml in coating buffer (11 mM Na_2_CO_3_, 35 mM NaHCO_3_, pH 9.6) and incubated overnight at 4°C. Plates were blocked with 2% [weight/ volume (w/v)] skim milk in PBS for 1 h at 37°C. Then, the plates were incubated with the serum samples diluted with 1°% (w/v) skim milk, 1°% [volume/volume (v/v)] Tween 20 in PBS, during 2 hours at 37°C. The antimouse IgG peroxidase conjugate (Sigma, St Louis, MO, USA) was added after and incubated 1 hours at 37°C. The reaction was developed with the substrate solution [52 mM Na_2_HPO_4_, 25 mM citrate, 1 mg/ml OPD, 0.1% (v/v) H _2_O_2_] for 15 minutes at room temperature. The reaction was stopped with 50 μl (3 M H_2_SO_4_ solution). Finally, the plates were read to 492 nm in a microtiter plate reader (Sensident Scan, Merck). Washes with 0.05% (v/v) Tween 20 in PBS solution were carried out between each step described below three to five times.

The IgG subclasses were evaluated using the kit ISO-2 Mouse Monoclonal Antibody Isotyping Reagents according to the manufacturer’s recommendations (Sigma-Aldrich).

Positive samples for antibody titers were detected using cut-off values of twice the optical density (OD) of negative controls (preimmune serum). OD values from samples were processed using an Excel program that is able to determine a value of titer plotting the OD values on the standard curve of known titers. This standard curve was included in each individual plate. Finally, the obtained results of total IgG and subclasses were represented as logarithm of geometric mean of titer (GMT) for each group of treatment (with a confidence interval of 95%). For the non-seroconverting sera, an arbitrary titer of 1:50 was assigned for statistical processing.

### ELISPOT Assay for determining Interferon-gamma (INF-g) Response

Preparation of Target and Effector Cells

The last immunization after 10 days, the spleens of 18 mice were aseptically removed and individual-cell suspensions were prepared. Erythrocytes were lysed after 5 minutes of incubation with 1.0 ml per spleen of 0.83% (w/v) NH_4_Cl. The cells were extensively washed with medium, diluted in complete medium RPMI 1640 (Gibco, USA), supplemented with 10% (v/v) fetal calf serum (FCS) (Gibco, USA), 2 mM glutamine, 2 mM pyru-vate, 50 mM 2-mercaptoethanol and antibiotics and counted. Meanwhile, H-2d mastocytome cells p815 were pulsed for 1 h at 37°C, 5% CO_2_ in complete medium with 10 mM S28-39 peptide (IPQSLDSWWTSL) from HBsAg (purified in the CIGB).^24^ After incubation, p815 cells were further incubated for another 15 min with mitomycin C (Sigma). They were extensively washed to avoid any trace of mitomycin C, and resuspended in complete medium for counting. The p815 cells without peptide were also treated as controls.

In vitro Restimulation of Primed CTL

After washing, the cells were counted and distributed in 25 cm^2^ flasks (Becton, Dickinson, England) at 2 × 106 cells per milliliter in 10 mL of complete medium, and stimulated with 5 μg/ml of peptide S28-39 during 4 days at 37°C and 5% CO_2_. Then, half of the total medium was substituted and new medium containing 20 UI/ml of IL-2 (CGEB, Cuba) was added. On day 7, the cells were collected and counted.

ELISPOT Assay

Nitrocellulose-backed 96-well, MAHA S45 plates (Milli-pore, France) were coated with 100 μl of 5 ug/ml murine IFN-g specific mAb R4-6A2 (Pharmingen, Becton Dickinson, England) overnight at 4°C, washed three times with PBS and blocked using complete medium at 37°C for 1 h. Two dilutions of freshly isolated (2 × 105 and 1 × 105) or restimulated splenocytes (104 and 5 × 104) and 1 × 105 p815-pulsed with the peptide S28-39 were incubated 20 hours at 37°C in 5% CO_2_. Splenocytes incubated with 2.5 ug/ml of concanavalin A (ConA) (Sigma, USA) were used as positive controls. Every group was controlled by the same number of wells incubated with unpulsed p815 cells as a negative control and the experimental controls of untreated mice.

After the 20 hours of incubation, the plates were washed three times with PBS and five times with PBS-0.05% (v/v) Tween 20, then 0.5 ug/ml of anti-IFN-g-biotin conjugated (antibody XMG1.2, Pharmingen, Becton Dickinson, England) was added and reacted at room temperature for 2 hours. Then, the wells were washed five times with PBS-0.05% (v/v) Tween 20, and peroxidase-labeled streptavidin (Sigma, USA) was added at 1:1000 dilution for 1 hour. The plates were washed again with PBS-0.05% (v/v) Tween 20 and PBS alone and the spot were developed by adding 3,3’-diaminobenzidine (3,3’,4, 4’-tetra-aminobiphenyl) tet-rahydrochloride (Sigma, USA) in 50 mM Tris-HCl, pH 7.4 with 0.3% (v/v) H_2_O_2_. After 15 min reaction was stopped with tap water, dried and the spot were counted under a dissection microscope (Zeiss, Germany). ELISPOT assay was conducted under re-stimulation with the S28-39 peptide. A positive response was considered by two times the level of spots of the negative control plus 10 spots.

### ELISA for Detection of Serum HBsAg

Detection of HBsAg in blood was carried out using a homemade standardized ELISA. Briefly, the solid phase was sensitized with the CB HepB.4 (CGEB Sancti Spiritus, Cuba) monoclonal antibody (10 ug/ml in 100 μl of coating buffer for 20 minutes at 50°C). Then, the plates were washed four fold with a 0.05% Tween 20 in deionized water. Subsequently, the plates were incubated with the serum samples diluted with 2% (w/v) skim milk during 1 hour at 50°C. After washing, 100 μl peroxidase-conjugated second antibody HRP-CB Hep1 (CIGB) was added to wells and incubated for 30 mins at 50°C. Finally, the plates were washed eight fold and the reaction was developed with the substrate solution for 10 min at room temperature. The reaction was stopped with 50 μl 3 M H_2_SO_4_ solution and the plates were read to 492 nm in a microtiter plate reader (Sensident Scan, Merck).

## HISTOPATHOLOGY

For each animal (18 HBsAg-Tg mice), the liver, kidneys, heart, spleen and lungs were macroscopically inspected and lesions scored under magnification. Further comparisons to control animals were carried out. Tissues samples were immediately fixed by immersion in 10% (v/v) buffered formalin and embedded in paraffin. Sections were cut at 5 μm and stained with hematoxylin and eosin (H&E) reagents.

## STATISTICAL PROCEDURES

GraphPad Prism version 4.00 statistical software (Graph-Pad Software, San Diego, CA, USA) was used to carry out statistical analyses. All titers were transformed to log_10_ for a normal distribution. The statistical treatment was carried out using the ‘F’ test to evaluate variance homogeneity followed by the student ‘t’-test, in case of two groups’ comparisons. For multiple groups comparisons, the results were analyzed using the program GraphPad Prism version 4.00 (GraphPad Software, USA), selecting one-way ANOVA and Newman Keuls test as parametric test, or Kruskal-Wallis and Dunns tests in case of non-parametric. A value of p < 0.05 was considered statistcally significant.

## RESULTS

The present study evaluated the immunogenicity of a novel therapeutic vaccine candidate under different conditions. The dynamic of the immune response in HBsAg-tg mice was characterized after subcutaneous or simultaneous mucosal-parenteral immunization during 25 administrations. In a second experiment was assessed the effect of increasing the antigen dose and the use of multiple mucosal immunization routes in the subversion of the tolerance to HBsAg.

### Effect of Simultaneous IN/SC Immunizations *vs* SC Immunization

Dynamic of the Anti-HBsAg IgG Response

**Fig. 1:**
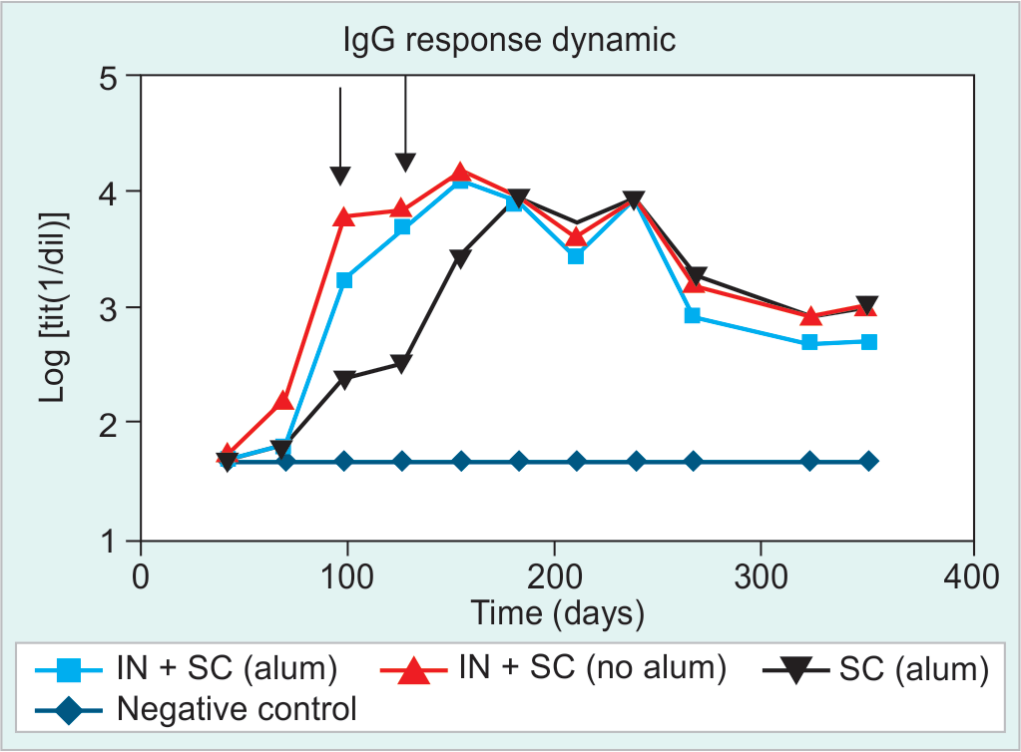
Kinetic of HBsAg-specific IgG response in sera. Immunization groups: (1) HBs/HBcAg (IN and SC), (2) HBs/HBcAg (IN) and HBs/HBcAg + Al(OH)_3_ (SC), (3) PBS 1x (IN) and HBs/ HBcAg (Sc), (4) negative control

**Fig. 2:**
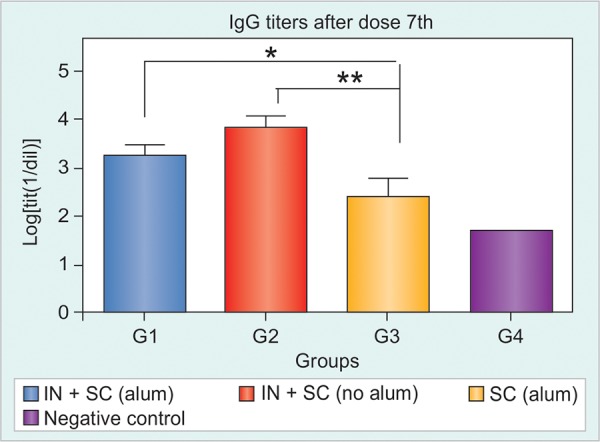
The HBsAg-specific IgG response in Tg mice after seven immunizations with: (1) HBs/HBcAg (IN and SC), (2) HBs/HBcAg (IN) and HBs/HBcAg + Al(OH)_3_ (SC), (3) PBS 1x (IN) and HBs/ HBcAg (SC), (4) negative control (*p < 0.05, **p < 0.01)

The HBsAg-specific IgG response was detected in HBsAg-tg mice only after the 5th dose, at very low frequencies. After further inoculations, it was demonstrated that groups immunized simultaneously by IN and SC routes (G1 and G2) induced stronger serum anti-HBsAg responses compared to group 3, immunized by SC route alone even when G3 had the same total dose for both antigens (Figs 1 and 2).

The study of the dynamic of Ab induction evidenced statistically significant differences after 7th and 9th doses (Fig. 2). A similar intensity of IgG response was reached for all immunized groups after dose 13th and all groups developed similar anti-HBsAg IgG response until the end of the study. It was not possible to find any difference between the groups that received the product simultaneously by IN and SC routes with or without alum (G1 and G2 respectively), although there was an initial trend in favor of G2 (Fig. 2).

During the last 5 to 10 immunizations it was possible to detect the effect of tg mice senescence on the anti-HBsAg-IgG response. No difference was found among the immunized groups regarding the dynamic of IgG reduction on time (Fig. 1).

Anti-HBsAg-IgG Subclass Response in Sera

**Fig. 3:**
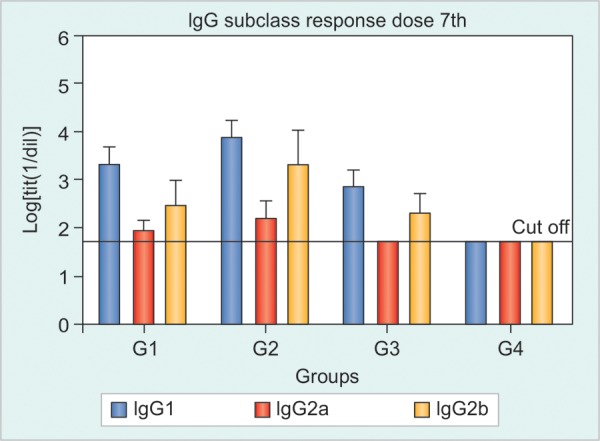
The HBsAg-specific IgG subclass patterns in Tg mice after seven immunizations with: (1) HBs/HBcAg (IN and SC), (2) HBs/ HBcAg (IN) and HBs/HBcAg + Al(OH)_3_ (SC), (3) PBS 1x (IN) and HBs/HBcAg (SC), (4) negative control

The IgG subclass responses (IgG1, IgG2a and IgG2b) anti-HBsAg were evaluated after 7, 11 and 15 immunizations. The IgG1 subclass was predominant in all groups compared to the IgG2a and IgG2b. No significant differences were observed among the groups, a trend of superiority in favor of the non-adjuvanted Group 2 was also observed after dose 7th, resembling the IgG response (Fig. 3). The groups immunized by the IN route evidenced a faster seroconversion to IgG2a and a stronger IgG2b response. The variability found in the study of IgG subclasses affected potential statistic differences among groups; however, it was clear that the inclusion of alum in the parenteral formulation did not improved the resulting immune response in any subclass as in general all subclasses had superior levels in the group 2 compared to group 1 (Fig. 3).

HBsAg Detection in Sera

**Fig. 4:**
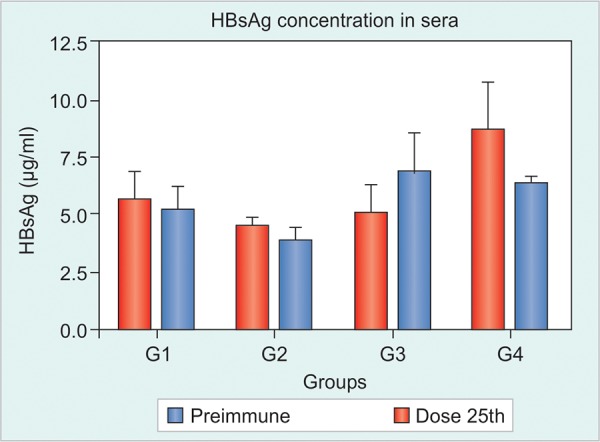
Detection of HBsAg in sera. Immunization groups: (1) HBs/ HBcAg (IN and SC), (2) HBs/HBcAg (IN) and HBs/HBcAg + Al(OH)_3 _(SC), (3) PBS 1x (IN) and HBs/HBcAg (SC), (4) negative control

Initial HBsAg concentration ranged between 5 and 10 ug/ml. It was not possible to detect any reduction in the serum levels of HBsAg as a result of the immunization protocol. The differences observed in the comparison before *vs* after were, in all cases, lower that the differences found in the negative control group (Fig. 4).

Anti-HBcAg IgG Response in Sera

As early as the third dose, anti-HBcAg antibody response was induced to a high level for all immunized groups (data not shown). No trend or statistical difference in the intensity of anti-HBcAg response was found.

### Effect of the Antigen Dose and the Simultaneous Use of Several Mucosal Immunization Routes

Anti-HBsAg Serum IgG Response

**Fig. 5:**
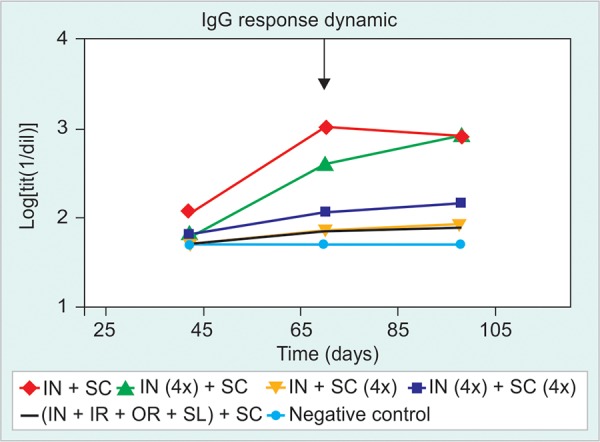
Kinetic of HBsAg-specific IgG response in sera. Immunization groups: (1) HBs/HBcAg 1x (IN and SC), (2) HBs/ HBcAg 4x (IN) and HBs/HBcAg 1x (SC), (3) HBs/HBcAg 1x (IN) and HBs/HBcAg 4x (SC), (4) HBs/HBcAg 4x (IN) and HBs/HBcAg 4x (SC), (5) HBs/HBcAg 1x (IN, IR, oral, SL) and HBs/HBcAg 1x (SC), (6) negative control

**Fig. 6:**
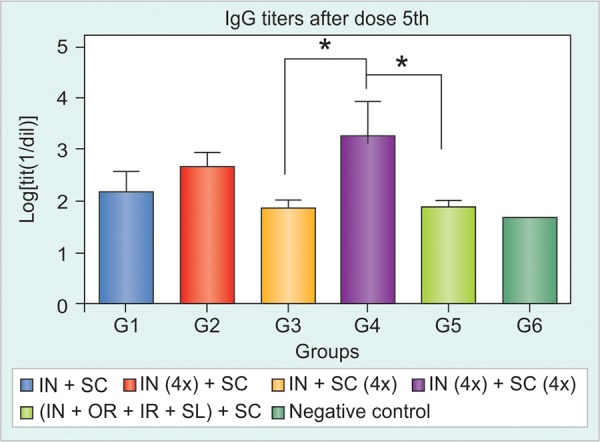
The HBsAg-specific IgG response in Tg mice after five immunizations with: (1) HBs/HBcAg 1x (IN and SC), (2) HBs/HBcAg 4x (IN) and HBs/HBcAg 1x (SC), (3) HBs/HBcAg 1x (IN) and HBs/ HBcAg 4x (SC), (4) HBs/HBcAg 4x (IN) and HBs/HBcAg 4x (SC), (5) HBs/HBcAg 1x (IN, IR, oral, SL) and HBs/HBcAg 1x (SC), (6) negative control (*p < 0.05)

In line with the results obtained in the previous immunization schedule, the HBsAg specific IgG responses were clearly detected after the 5th immunization for most immunized groups. However, the group that received the highest antigen dose by IN and SC routes (G4), had a high frequency of seroconversion to anti-HBsAg IgG after the 3rd immunization. This group (G4) induced a higher IgG response compared to the rest of the groups after the fifth dose, reaching statistic signification (p < 0.05) compared to G3 subcutaneous enriched dose, and group 5 immunized by different mucosal routes (Fig. 5). The groups receiving 20 μg by IN route (groups 2 and 4) induced the higher responses; conversely, the increase in the dose by the SC route (G3) did not appear to improve the resulting anti-HBsAg-IgG response. After dose 7th the immune response generated by Groups 2 and 4 was similar, but still superior to the immune response induced by the rest of the groups (Fig. 5). There was no enhancing effect on anti-HBsAg-IgG response by splitting the dose by four different mucosal routes group 5 (Figs 5 and 6).

HBsAg Specific IgG Subclass Responses

**Fig. 7:**
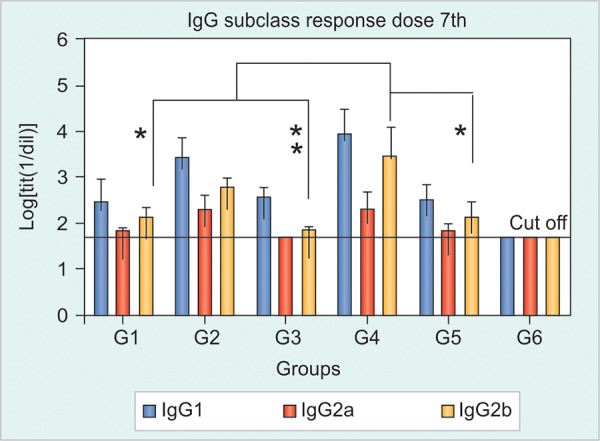
HBsAg-specific IgG subclass patterns in Tg mice after seven immunizations with: (1) HBs/HBcAg 1x (IN and SC), (2) HBs/HBcAg 4x (IN) and HBs/HBcAg 1x (SC), (3) HBs/HBcAg 1x (IN) and HBs/ HBcAg 4x (SC), (4) HBs/HBcAg 4x (IN) and HBs/HBcAg 4x (SC), (5) HBs/HBcAg 1x (IN, IR, oral, SL) and HBs/HBcAg 1x (SC), (6) negative control (*p < 0.05)

Serum IgG subclasses followed the same pattern found in the first experiment. Anti-HBsAg IgG1 was the predominant subclass. The pattern of intensity found in the levels of total IgG was repeated in the case of IgG subclasses. After dose 7th the titer of the IgG2b subclass was significantly superior in the group immunized with the higher dose (G4) compared to groups 1 and 5 (p <0.05) and was very significant (p < 0.01) regarding group 1 (Fig. 7).

HBsAg Detection in Sera

**Fig. 8:**
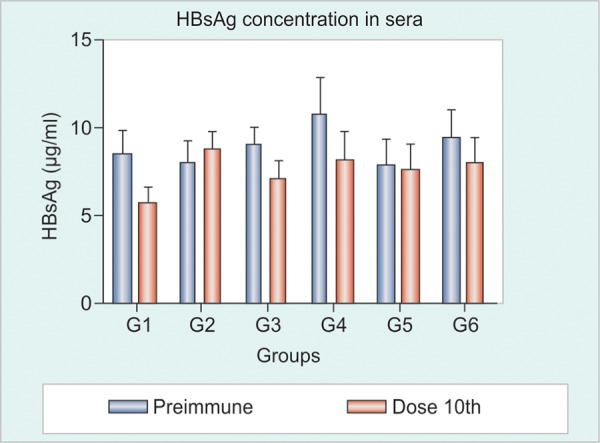
Detection of HBsAg in sera. Immunization groups: (1) HBs/ HBcAg 1x (IN and SC), (2) HBs/HBcAg 4x (IN) and HBs/HBcAg 1x (SC), (3) HBs/HBcAg 1x (IN) and HBs/HBcAg 4x (SC), (4) HBs/ HBcAg 4x (IN) and HBs/HBcAg 4x (SC), (5) HBs/HBcAg 1x (IN, IR, oral, SL) and HBs/HBcAg 1x (SC), (6) negative control

In this second experiment, it was found no reduction in any of the extractions up to the 10th dose, in line with the results found in the first experiment (Fig. 8). In general, all mice express HBsAg in high levels, between 5 and 10 ug/ml.

HBsAg-specific Secretion of Gamma-interferon Response by ELISPOT Assay

Mice immunized with 5 μg of each antigen by IN and SC routes were studied after 3, 5, 7 and 10 immunizations to explore if the timing of cellular response induction was associated to the start of the humoral immune response. As a result, it was possible to detect a positive HBsAg-specific immune response directed against the S_28-39 _CD8+ T cell epitope only after the fifth dose, the positive response was also detected at dose 10. Quantitative studies are required to compare different approaches of immunization and formulations.

### Histopathological Studies

After 25 inoculations of NASVAC, no histological damage was observed in the analyzed organs related to the immunization of formulations containing the HBs/HBcAg and HBs/HBcAg/alum. The liver changes found in all HBsAg-Tg mice used in these experiments (immunized and control) were previously reported.^22^

## DISCUSSION

The HBsAg-tg mice have been previously used as a model of immunotolerance to evaluate several therapeutic vaccine candidates.^25,26^ In the present article, a therapeutic vaccine candidate based in the mixture of HBsAg and HBcAg antigens is evaluated in HBsAg-Tg mice to explore the possibilities of optimizing the resulting immunity as well as the histopathological damage associated to product administration.

A first experimental setting evaluated the effect of the adjuvant aluminiun hydroxide frequently used in HBsAg-based preventive vaccines, on the resulting immune response to NASVAC. The most potent and faster immune response was found in the group immunized without alum (G2) compared with the corresponding group (G1), receiving the same HBsAg dose by simultaneous IN and SC immunization containing alum in the parenterally administered formulation. The result was also confirmed during the study of the Ab subclasses. Taken together, these results suggest that there is no additional benefit on using alum under the immunization conditions. As it is expected that future clinical trials will involve a high number of inoculations in order to optimize the effect of stimulating specific anti-HBV immunity on the viral load, avoiding alum in the injected formulation will greatly improve the safety profile of the treatment.

Alum has been related to several adverse effects,^27,28^ its absence would provide space for the evaluation of multiple immunization schedules. In addition, alum is a Th2 adjuvant;^28,29^ the absence of alum will contribute to the reinforcement of the Th1 pattern, which is related to HBV control by the immune system after acute or chronic infections. The lack of effect found on the Ab titers under the study conditions suggest that the most important function expected from alum can be achieved according to the proposed schedule of treatment and in summary it is not necessary.

Previous results in non-tg Balb/C mice have shown that after IN/SC schedules of immunization mice treated with parenteral formulations of NASVAC containing alum develop a lower IFN-g secreting CD8+ T-cell response compared to the corresponding treatment with a parenteral formulation without alum.^30^

In vaccine studies, the HBcAg has shown to be a potent immunogen even without adjuvants.^10,11^ This particle has the ability to act as a potent B-cell activator, enabling activated B cells to work efficiently as primary APCs.^31^ Preclinical studies in mice with the combined HBsAg and HBcAg nasal vaccine candidate showed a high mucosal (nasal) immunogenicity of full length HBcAg and the immunoenhancing activity on coadministered HBsAg.^19,32,33^

In the specific case of chronic HBV therapeutic vaccination, the nasal administration of a combination of HBsAg and HBcAg should help in the generation of more potent and generalized anti-HBV responses in infected patients and can be used as an adjunct to parenteral therapeutic vaccination with standard or enhanced HBV vaccines.^19^ The results support the use of IN/SC combination also in terms of their superior immunogenicity due to their faster induction of HBsAg specific antibodies in sera. This result evidenced that the IN route is important for improving anti HBsAg immune response in conditions of immune tolerance.

The immune response was not able to control HBsAg even after 25 administrations (1 year of treatment) under the assayed conditions. It has been previously reported by Akbar et al the control of the HBsAg in an important percentage of HBsAg-Tg mice after 8 months of treatment (using HBsAg in Freund’s complete adjuvant). However, it should be taken into account the differences between both animal models. In the present study, mice express HBsAg in serum concentrations up to 20 ug/ml and in the previous at levels below 1 ug/ml.^34^

The generated HBcAg specific immune response was strong and high for all groups immunized after 3 doses, in accordance with the immunological characteristic reported for this antigen.^35^

Several organ samples were taken for histological analysis. No histological damage was observed in the organs analyzed. All histological manifestation found in tg-mice organs have been previously reported during the characterization of the model.^22^

A second experiment was designed to evaluate the effect of dose increase by parenteral or IN route as well as the effect of splitting the IN dose by several mucosal immunization routes, using the same formulation.

The results suggest that a balance for increasing doses by both routes is important as the preliminary result evidenced a different effect when the increase is through the nasal compared to the SC route. An additional indication of the role of the IN route in tolerance subversion compared to SC route.

The increase in the dose could be a way to optimize the process of tolerance subversion in the setting of therapeutic vaccination. A similar approach of increasing the dose has been previously attempted in low responders to preventive vaccination with positive results; this is the case of patients with unrelated liver diseases, under hemodialysis or suffering immunodepressions. The split in the dose of the immunogen by different mucosal routes did not improve the resulting immune response.

ELISPOT assays conducted in tg mice (summarized in Table 1) evidenced that the cellular immune response parallels the humoral immune response on time, confirming the dynamic of tolerance subversion on both arms of the immune system.

Another characteristic of the cellular immune response is the higher frequency of female tg mice with positive response by ELISPOT compared to male sex (Table 2). Future studies should select female mice (or only same sex mice) in order to homogenize results as the immune subversion of tolerance in male mice was poor according to their much reduced cellular response. Interestingly, male mice express a significantly higher HBsAg concentration compared to female mice (personal communication).

**Table 1:** **Table 1:** Dynamic of positive ELISPOT response in HBsAg-Tg mice immunized with the formulation under study according to the schedule: HBs/HBcAg (IN) and HBs/HBcAg + Al(OH)_3_ (SC) during 10 administrations. Mice were immunized every 14 days, and spleen cells were isolated 10 days after the 3rd, 5th, 7th and 10th doses

		*Doses and percentage of* *positive responses*							
		*3rd*		*5th*		*7th*		*10th*	
HBs/HBcAg (IN) and HBs/HBcAg + Al(OH)_3_		0/3		1/3		3/3		3/3
(SC)		(0%)		(33%)		(100%)		(100%)	

**Table 2:** **Table 2:** Female *vs* male percentage of positive ELISPOT response in HBsAg-Tg mice immunized during the second experiment and HBsAg concentration in sera assessed at preimmune extraction

		*Male*		*Female*	
Percentage of positive response by ELISPOT		1/9 (11%)		9/10 (90%)	
Conc. HBsAg (μg/ml) (time 0)		11.40		5.74	

In both experiments, there was no reduction in HBsAg concentration in the sera even when there was high-anti-HBsAg production in a few mice (Figs 4 and 7). HBsAg Tg mice model express the HBsAg at very high serum levels, usually more than 5 ug/ml (Table 2), and it is produced by several organs of the body, suggesting a strong central and peripheral tolerance also.

The administration of the vaccine candidate up to 25 times at 14 days interval evidenced a safe toxicological profile. The results of the histopathological evaluation further support the results found during previous pre-clinical toxicology studies.^36^
